# Twenty‐year experience with macro‐area school screening for andrological disease in paediatric age

**DOI:** 10.1111/and.14209

**Published:** 2021-08-11

**Authors:** Nicola Zampieri, Simone Patanè, Francesco Saverio Camoglio

**Affiliations:** ^1^ Pediatric Surgery Unit Woman and Child Hospital Azienda Ospedaliera Universitaria Integrata University of Verona Verona Italy; ^2^ Pediatric Fertility Lab Department of Surgery Dentistry, Pediatrics and Gynaecology University of Verona Verona Italy

**Keywords:** adolescence, children, infertility, varicocele

## Abstract

Varicocele, phimosis and undescended testes are the most frequent andrological diseases in paediatric age; varicocele and undescended testes are primary causes of male infertility and the interests of research about these conditions have changed in the last years. The aim of the study was to report our experience after 20 years of macro‐area school screening between 2000 and 2020. Data about school screening were reviewed and analysed. Subjects aged between 11 and 14 years underwent andrological visit. During the study period, three main andrological screenings were performed into our macro‐area. The distribution of cohorts was different among the screenings. Among andrological diseases, varicocele diagnosis increased especially in the last 10 years. Phimosis was diagnosed less respect the first screening (2000–2001), while at present there were no cases of undescended testes. Our experience reported some interesting data, especially for the higher incidence of varicocele detected on two consecutive school screening; our results demonstrate also the importance and the preventive role of andrological check‐up also in paediatric age and adolescence, to reduce the incidence of those diseases affecting the fertility potential.

## 1 INTRODUCTION

Many of the andrological diseases that occur in adulthood have their origins before the age of 18 years and sometimes even in the period of gestation. In fact, the male gonad is extremely susceptible to external insults—a sensitivity starting in the gestational period and lasting until puberty (Jacobson & Johnson, 2017; Zampieri & Camoglio, 2020).

It is therefore essential to have an andrological evaluation during childhood to observe abnormalities in the genital organs that occur very early such as alterations affecting the penis or abnormal positions of the testicle and to highlight risk factors for the subject's andrological and sexual health. During puberty, it is useful to monitor the timing of the onset of pubertal and testicular development and to evaluate the possible presence of gynaecomastia and testicular hypotrophy or varicocele (Cannarella et al., 2019; Zampieri, 2021), for the latter pathology is especially widespread among young people.

Even in this phase of life, the assessment of risk factors such as those related to lifestyles is of fundamental importance for primary prevention purposes and to provide adequate information to adolescent male and their families. Puberty corresponding to the achievement of reproductive and sexual maturity (Gorelick & Goldstein, 1993; Levinger et al., 2007; Olana et al., 2018; Zampieri & Cervellione, 2008): Preserving the genital and sexual health of young people also means protecting their fertility.

Young people in many schools and youth aggregation centres in the area demonstrated great interest and participated enthusiastically in various medical projects. If, on the one hand, the projects have represented epidemiological tools and screening for early identification of various andrological diseases, from young people's point of view, it has been understood as valid tools for information on topics difficult to deal with. In general, the update campaigns should also involve other doctors in the area in order to create or strengthen existing networks of care and enhance the local–regional experiences that have been already started. The purpose of this study is to report the results of the 20‐year andrological screening campaign within our macro‐area.

## 2 MATERIALS AND METHODS

Based on the result of the first screening performed between 2000 and 2001 (Zampieri and Cervellione, 2008), further 2 screenings were performed in 2010 (January–May) and in 2020 (January–March, September–October). Compared with the first screening that included two phases, during these two screenings, we focused mainly on the first phase, namely the epidemiological study with the aim of identifying the prevalence and incidence of major andrological diseases (varicocele, phimosis, hydrocele, cryptorchidism) in the 11–14 age group. During the 2020 screening, only periods outside of quarantine and periods during which it was allowed for patients to go to the hospital were considered.

### 2.1 Screening programme

Through an email list, all the institutes that had joined the first screening were contacted; the schools that joined the project made a list of participants. The participants were contacted, and a visit to the paediatric fertility laboratory was organised. The project was approved by the IRB (PFL 9/2020).

All patients, after giving their oral and written consent, were checked for andrological diseases at our hospital.

Patients were examined in the standing position: their testes were assessed for varicocele, hydrocele and cryptorchidism, and their penises were examined for phimosis.

All patients underwent testicular Doppler velocimetry for spermatic vein reflux. Testicular hypotrophy was defined as a decreased testicular volume of at least 20% (which is considered the minimum palpable testicular difference) compared with the contralateral testis. Those having testicular hypotrophy underwent testicular ultrasound to quantify the correct volume.

Varicocele was graded according to the Dubin and Amelar clinical classification. The testicular Doppler classification of Hirsh for vein reflux was applied (Zampieri et al., 2008).

Puberty was classified as 1 of 2 categories, including prepubertal‐absent pubic hair with infantile genitalia (grade I) and pubertal‐initial or complete development of pubic hair and genitalia (grade II). We decided to simplify pubertal development classification, not using Tanner's staging, to avoid differences in interpretation and thus facilitating patients’ classification.

Clinical data were transferred to a database.

### 2.2 Data interpretation

After collecting data, a comparison was made with the screening of 2000–2001. Data regarding the population and the number of students in the age range considered were searched through the website of our city in order to compare them with the incidence of individual pathologies.

### 2.3 Statistical analysis

Patient data were recorded in an electronic database; Student's *t* test was used to compare continuous variable and chi‐square was used to compare categorical variables using SPSS Chicago IL, USA, 16.0 for Windows; a value of *p* < .05 was considered significant.

## 3 RESULTS

During the study period (2000–2020), the population of the province remained essentially stable (Table 1). The number of adolescents between the ages of 11 and 14 was also essentially unchanged. For a total of 125 schools in the macro‐area, 97 schools joined the project in 2010 and 59 schools in 2020.

**TABLE 1:** Patients distribution and characteristics

Screening programme	Varicocele	Phimosis	Other	*p* value
2000–2001 4,186 students 2,107 screened	609 (28%)	452 (20%)	Hydrocele 19(0,9%) undescended testes 13 (0,6%)	
11,2 ± 0,3 11,5 ± 1,4 13,3 ± 0,6	G I−332 (54%) G II−143 (23%) G III−134 (22%)	13,6 ± 0,1		
2010–2011 4,644 students 1,998 screened	699 (34%)	356 (17%)	Hydrocele 4(0,2%) undescended testes 3 (0,1%)	
11,1 ± 0,9 12,4 ± 1,2 13,4 ± 0,4	G I−227 (32%) G II−376 (53%) G III−96 (14%)	12,4 ± 1,3		
2020 4,685 students 497 screened	212 (41%)	52 (10%)	0	
11,6 ± 0,3 12,3 ± 1,1 12,6 ± 0,3*	G I−54 (25%) G II−99(46%) G III−59 (27%)*	11,6 ± 0,2*		*p* = .02 *p* = .03

Brief summary of 2000–2001 screening data: from a male school population of 4,186 children and adolescents, 2,107 boys aged between 10 and 16 joined the study. A varicocele was diagnosed in 609 boys (28%) of whom 42% were between 10% and 12%, 32% between 12% and 14% and 26% between 14 and 16. Varicocele was grade I to III in 332, 143 and 134 cases respectively. During the screening, 593 subjects (28%) had phimosis, 46 (2.1%) had hydrocele, and 9 (0.4%) had undescended testes. The mean age of patients with phimosis was 13,2 ± 1,1 years. Among pubertal status, 622 (29%) were grade II while the rest was considered as grade I.

### 3.1 Screening 2010–2011

During 2010, from a male school population of 4,644 children and adolescents 1,998 boys (12 ± 1,2 years) joined the study.

A varicocele was diagnosed in 699 boys (34%) of whom 40% were between the ages of 11 and 12 and the rest, over 12. There were 227 (32%) grade I, 376 (53%) grade II and 96 (14%) grade III patients.

Patients’ age distribution per varicocele grade was as follows: 11,1 ± 0,9 years for grade I; 12,4 ± 1,2 years for grade II; and 13,4 ± 0,4 years for grade III.

During the screening, 356 subjects (17%) had phimosis, 4 (0.2%) had hydrocele, and 2 (0.1%) had undescended testes. The mean age of patients with phimosis was 12,4 ± 1,3 years.

Among pubertal status, 527 (26%) were grade II while the others were considered as grade I.

### 3.2 Screening 2020

During 2020, from a school population of 4,685 children and adolescents 497 (12 ± 0,9 years), boys were joined the study.

A varicocele was diagnosed in 212 boys (41%) of whom 43% were between 11 and 12, and the rest were over 12 years of age. There were 54 (25%) grade I, 99 (46%) grade II and 59 (27%) grade III subjects. The patients’ age by degree of varicocele was distributed as follows: grade I 11,6 ± 0,3 years, grade II 12,3 ± 1,1 years and 12,6 ± 0,3 years for grade III.

During the screening, 52 subjects (10,4%) were diagnosed with phimosis; no patients had hydrocele or undescended testes. The mean age of patients with phimosis was 11,6 ± 0,2 years.

Among pubertal status, 125 (25%) were grade II while the rest was considered as grade I.

What we can report observing the data of the two screenings performed about 10 years apart is that over time, the percentage of patients with varicocele has increased and the diagnosis is performed earlier than in the previous decade (*p* < .05). Those patients with grade III varicocele were younger respect to those in the first screening.

This finding seems to be independent of the pubertal stage at diagnosis. Through the study of the type of venous reflux with Doppler ultrasound, no differences were noted during this 20 years of screening. As also demonstrated in other studies, the clinical grade does not correspond to the degree of venous reflux.

Compared with the previous screening, a prevalence reduction of diseases was also noted. This can be explained by the activation of several prevention campaigns, which was proposed both by schools and by paediatricians (*p* < .05).

We have neither considered the number of patients nor the percentage of patients who underwent surgery for varicocele and testicular hypotrophy over the years because the third screening has begun only recently. However, we can report that the percentage of testicular hypotrophy associated with varicocele at the time of the first visit has always remained homogeneous over the years: 17% in the first screening and 21% and 22% in the second and third screening respectively.

## 4 DISCUSSION

The worrying demographic decline in developed countries is well known, and the increase in andrological diseases has certainly contributed to this decline. In 40%–50% of the cases, the infertility lies with the male. However, while women are followed and studied in depth in the gynaecological field, many males decide to undergo a check‐up of their reproductive system only if they find difficulty in fathering a child. Often even at this point, they are not always subjected to an andrological check‐up. Most of the time, the couple is sent to medically assisted procreation centre to receive exclusive gynaecological treatment. In order to diagnose andrological diseases early, it is necessary to implement an organised health plan. The purpose of an andrological screening in a juvenile population is to observe any clinically detectable diseases/pathologies at the end of pubertal development which potentially carries the risk of impeding future fertility. These potential risks are conditions of varicocele, hypogonadism, genital malformations, etc. Certainly, varicocele and undescended testes represent the most frequent eventualities that can occur during the peri‐pubertal period. Sexual health is very important, not only for the quality of life but also because it is a mirror of general health, especially in old age. Prevention must start at a young age: preserving fertility potential and sexuality with a proper lifestyle means preserving overall health. An andrological examination and therefore screening in adolescence can help doctors diagnose and solve many early pathologies that could compromise adult sexual health such as phimosis, erectile dysfunction and other functional problems, sexually transmitted diseases, and problems such as testicular tumours and common testicular diseases. (Jensen et al., 2018)(Zampieri & Cervellione, 2008)(Zampieri et al., 2008).

Preventive interventions in andrology should therefore have the following main objectives: to provide all the information necessary for the prevention of the most frequent diseases and the most serious diseases of andrological relevance and to identify as early as possible the most important and frequent diseases of the male reproductive system.

During this period, within our macro‐area, we have organised together with schools and paediatricians different screening projects, for example on diet, incidence of obesity and sexuality. The advantage of continuous contact with young people was appreciated during pubertal development. Currently, thanks to the screening policies, we have reduced the number of surgical interventions for phimosis; patients with varicocele and testicular hypotrophy were treated earlier, and thanks to the meetings in schools, we have made people understand the importance of performing the semen analysis to monitor the fertility potential. At our department, patients can perform the semen analysis already after the age of 16 years.

Among our important results, some limitations emerge from our study:

1. The study sample, although large, may not be homogeneous with respect to the samples of previous screenings.
2. The population selected and visited in these 3 different screenings came from the same province; however, different factors may have influenced especially the increased incidence of varicocele over 20 years such as sports, food, environment, residence within the same province.
3. During the first screening, we noticed that the incidence of varicocele was higher in boys who lived in the city than in boys who lived in the suburbs (unpublished data). This assessment was not considered during the 2010 and 2020 screenings.
4. Since the investigator is always the same, after 20 years, the dexterity and the diagnostic accuracy have surely improved, especially for varicocele and testicular volume, thus generating an increase in relative diagnosis.

From the results of the study, however, it is possible to deduce that the increased incidence of varicocele inevitably correlates with the literature on adult andrology: varicocele is therefore a potential cause of impaired fertility in males. It should also be noted that the average age at diagnosis especially of the high grades (II and III) decreased in the last 20 years. However, the results of the screening also show important data: over the years, the incidence of phimosis has decreased thanks to the continuous dialogue between the hospital and paediatricians in the macro‐area. These data are also significant for the other two most common pathologies: hydrocele and undescended testes essentially disappeared. With regard to these pathologies, they certainly do not affect fertility like varicocele; however, they are the main causes of infections, such as balanitis xerotica obliterans (Nguyen & Holland, 2020; Punjani et al., 2021), testicular pain and alteration of the seminal fluid in adults.

At present, thanks to the results of this screening, together with the Italian National Health system, we have created an andrological medical sheet to be filled in with paediatricians as several controls from birth to 16 years. In this way, patients are not lost and do not arrive late to the intervention. For example, all males must be checked at 6 months and at 12 months of age to assess testes position, at 1 year and 3 years of age they are checked for phimosis and at 10 and 12 years of age they are visited to assess the presence of varicocele. Future treatments follow the normal clinical practice with the use of cortisone for phimosis, and surgery in cases of testicular hypotrophy associated with varicocele.

Young adolescents are not followed as much as their female peers, and this problem is also reported in other screening experiences. Screening campaigns therefore serve not only to diagnose diseases early, but also to make adolescents aware that sexual health and fertility potential are also influenced by genital health.

## 5 CONCLUSION

It is important to organise andrological screening, focusing on truly paediatric age groups; many of the pathologies can be diagnosed already by 12 years of age. The sexual and physical health of young people will then be the health of the adult, so it is necessary to avoid generating infertility.

Our study shows how prevention is important and how the clinical characteristics of adolescent patients are modified over time.

## Data Availability

The data that support the findings of this study are available from the corresponding author upon reasonable request.
